# A case of small in situ perihilar cholangiocarcinoma incidentally accompanied by benign bile duct stricture after open cholecystectomy

**DOI:** 10.1186/s40792-019-0745-z

**Published:** 2019-11-09

**Authors:** Takashi Maeda, Tomoki Ebata, Yukihiro Yokoyama, Tsuyoshi Igami, Takashi Mizuno, Junpei Yamaguchi, Shunsuke Onoe, Nobuyuki Watanabe, Masato Nagino

**Affiliations:** Division of Surgical Oncology, Department of Surgery, Nagoya University Graduate School of Medicine, 65 Tsurumai-cho, Showa-ku, Nagoya, 466-8550 Japan

**Keywords:** Benign bile duct stricture, Carcinoma in situ, Perihilar cholangiocarcinoma, Aspiration bile cytology

## Abstract

**Background:**

In situ cholangiocarcinoma is difficult to detect by imaging studies. Thus, cholangiocarcinoma is rarely resected with a preoperative definitive diagnosis, especially nonpapillary flat type in situ carcinoma, which is extremely rare.

**Case presentation:**

A 70-year old man was diagnosed with gallbladder cancer and received open cholecystectomy with lymphadenectomy at a local hospital. Histologically, the tumor was localized in the mucosal layer, and no lymph node metastases were found. Three months later, hilar bile duct stricture due to delayed bile duct ischemia was found. Then, biliary drainage was performed with endoscopic biliary stenting. Three months later, the patient experienced cholangitis with septic shock, and percutaneous transhepatic biliary drainage (PTBD) into the left intrahepatic bile duct was performed. Unexpectedly, the aspiration bile cytology of the PTBD catheter showed malignant cells, and the patient was referred to our clinic for possible surgical treatment. According to additional studies, the hilar bile duct stricture was 3 cm in length. None of the imaging studies detected malignant cells in the bile duct around the hilar stricture. The left portal vein was obstructed due to inadvertent puncture of the PTBD. No findings indicated cholangiocarcinoma. We performed left hepatectomy with caudate lobectomy and extrahepatic bile duct resection. The postoperative course was uneventful. In the final pathology, flat type in situ carcinoma was found at the confluence of the right and left hepatic ducts, which was distant from the biliary stricture.

**Conclusions:**

When a tumor is undetectable but cytology is positive, in situ cholangiocarcinoma may exist; thus, surgery should be carefully considered.

## Background

In situ cholangiocarcinoma, i.e., epithelial carcinoma without submucosal invasion, is asymptomatic and very difficult to detect by multidetector-row computed tomography (MDCT) or direct cholangiography. Thus, this type of carcinoma is rarely resected with a preoperative definitive diagnosis, especially, nonpapillary flat type in situ carcinoma, which is extremely rare.

Here, we present a case of flat type in situ perihilar cholangiocarcinoma that was incidentally accompanied by hilar bile duct stricture after open cholecystectomy. Preoperative diagnostic imaging studies did not identify the lesion, and only aspiration bile cytology showed a positive result.

## Case presentation

**Fig. 1:**
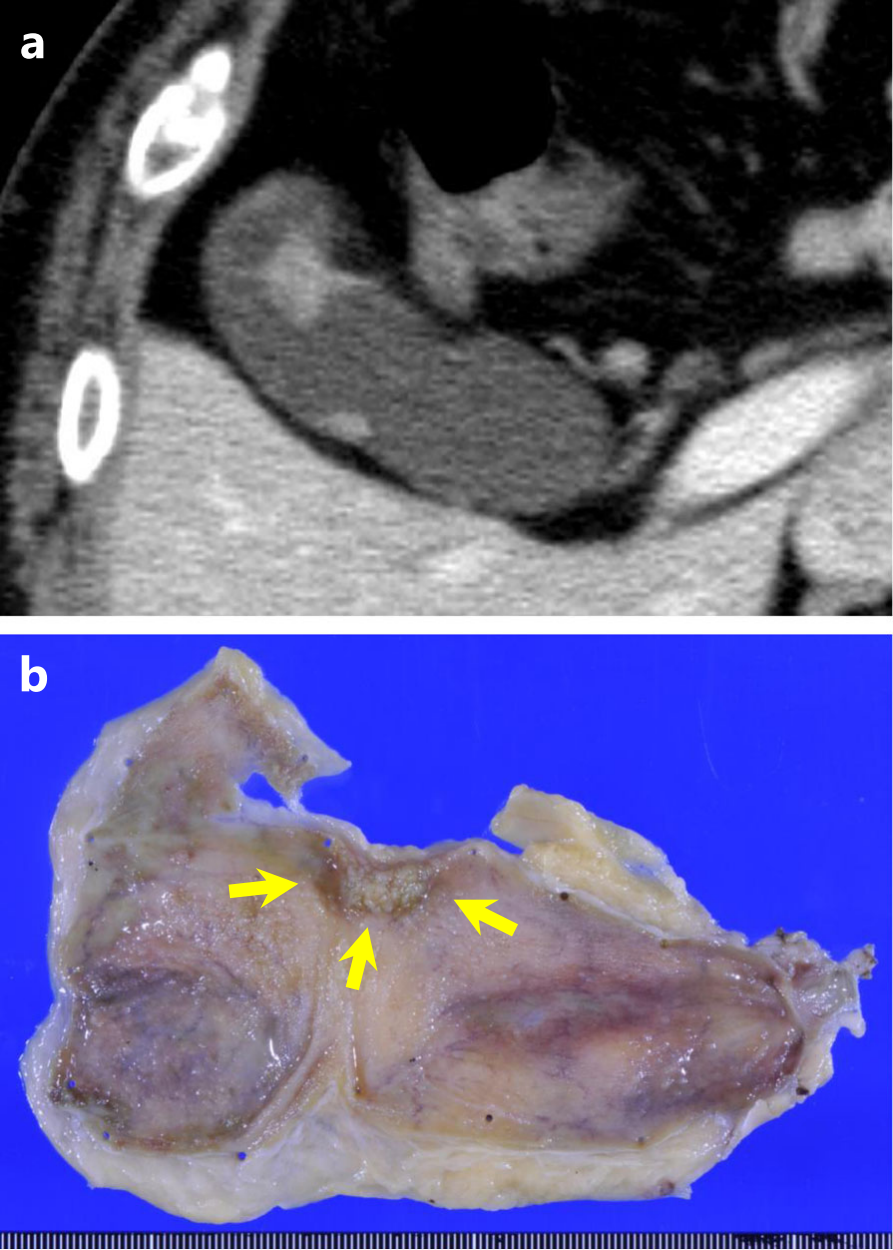
Images of gallbladder cancer. **a** Computed tomography revealing a papillary tumor in the gallbladder. **b** Resected specimen. The yellow arrows indicate a papillary tumor

**Fig. 2:**
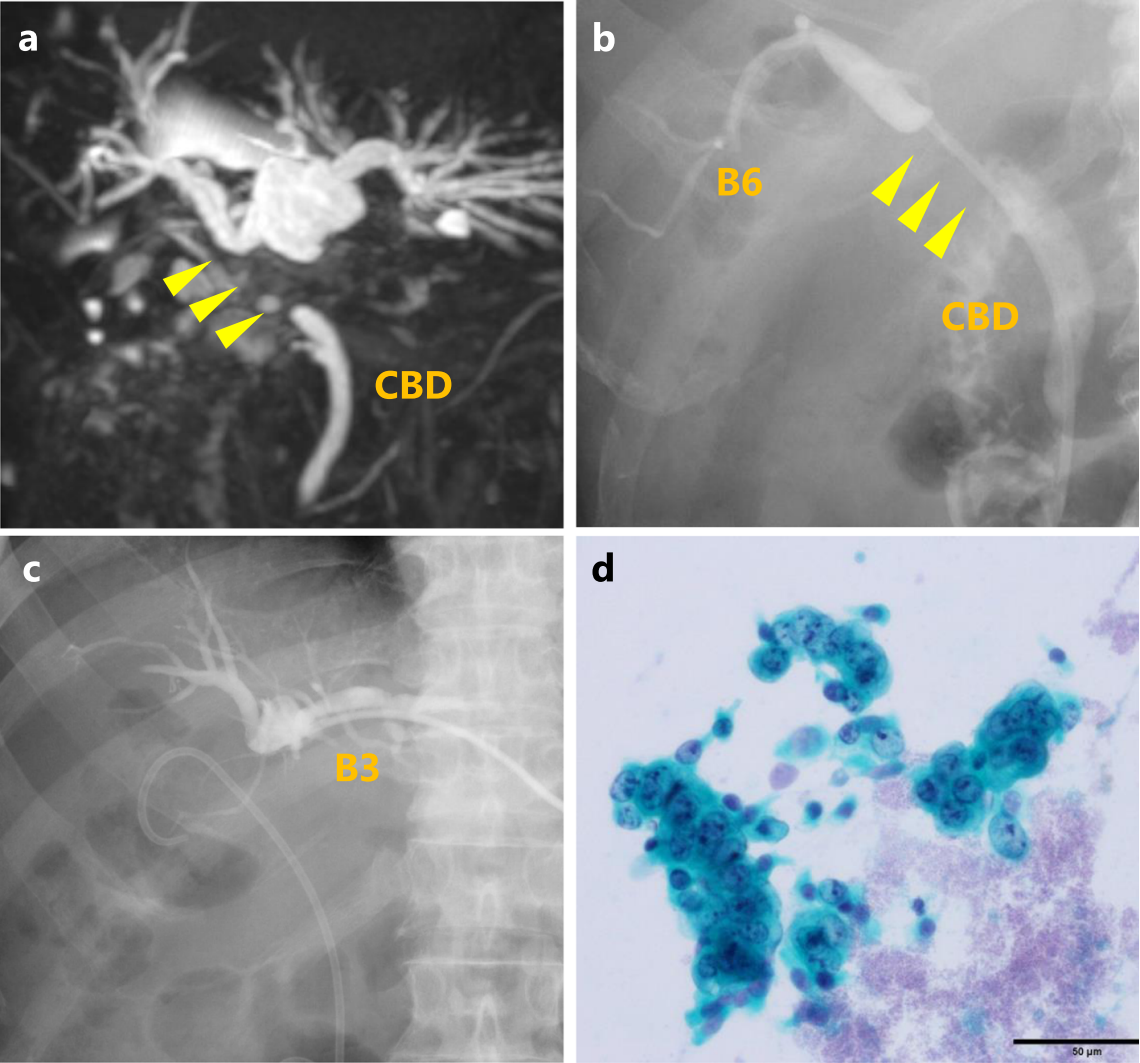
Images of the bile stricture. **a** MRCP showing a hilar bile duct stricture (yellow arrowheads). **b** Endoscopic biliary stent placed in the right posterior inferior bile duct (B6). **c** Percutaneous transhepatic biliary drainage into the left lateral anterior bile duct (B3). **d** Aspiration bile cytology from the PTBD showing malignant cells. MRCP, magnetic resonance cholangiopancreatography; CBD, common bile duct; PTBD, percutaneous transhepatic biliary drainage

A 70-year old man was diagnosed with gallbladder cancer (Fig. 1a) and received open cholecystectomy with lymphadenectomy of the hepatoduodenal ligament at a local hospital. The final pathology showed that the tumor was a moderately differentiated adenocarcinoma with an invasion depth of the mucosal layer. No lymph node metastases were found, and all of the surgical margins were negative (Fig. 1b). The patient was discharged from the hospital, but 3 months later, magnetic resonance imaging (MRI) showed hilar bile duct stricture with intrahepatic biliary dilatation (Fig. 2a), probably due to delayed bile duct ischemia caused by lymphadenectomy. Then, biliary drainage was performed with endoscopic biliary stenting (Fig. 2b). Three months later, the patient experienced cholangitis with septic shock, and percutaneous transhepatic biliary drainage (PTBD) into the left intrahepatic bile duct was performed (Fig. 2c). Unexpectedly, the aspiration bile cytology from the PTBD catheter showed malignant cells (Fig. 2d). Percutaneous transhepatic cholangioscopy (PTCS) was performed via the sinus tract of PTBD, but the examination failed to detect any malignant lesions in the biliary tree. The patient was referred to our clinic for possible surgical treatment.

**Fig. 3:**
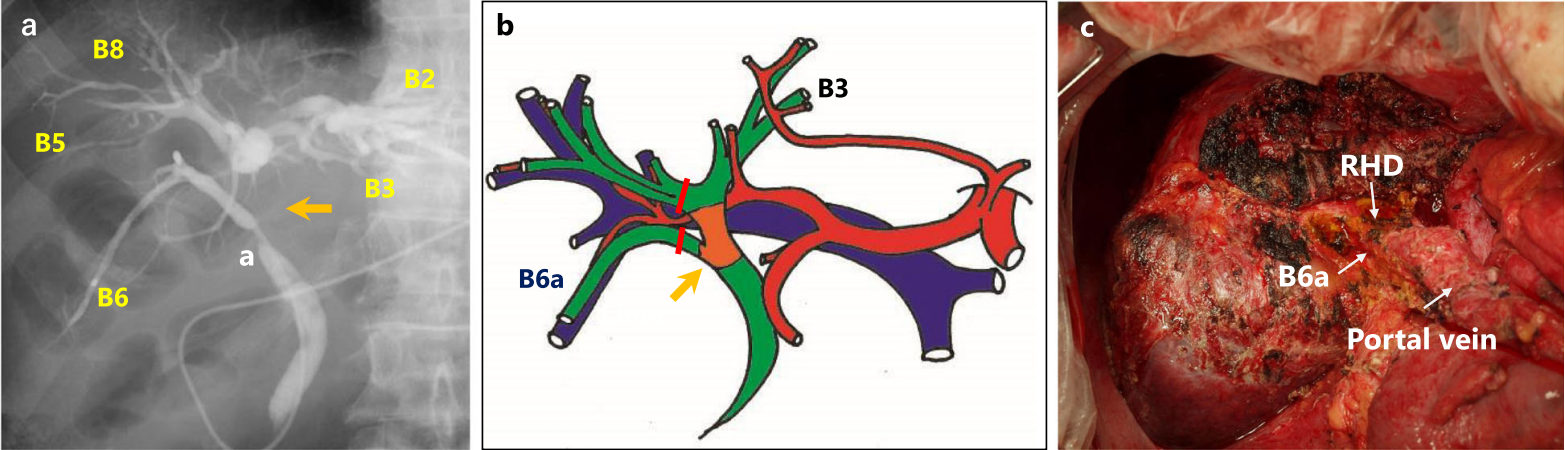
Cholangiogram, preoperative schema, and operative picture. **a** Cholangiogram showing a hilar bile duct stricture (orange arrow). **b** Preoperative scheme. The planned surgery was left hepatectomy with caudate lobectomy. **c** Left hepatectomy with en bloc resection of the caudate lobe and extrahepatic bile duct was performed as scheduled. The right hepatic duct (RHD) and the right posterior inferior bile duct (B6a) were reconstructed separately

After admission, the patient’s cholangiograms were re-evaluated (Fig. 3a, b). The right posterior inferior bile duct (B6) was the infraportal type and joined the common hepatic duct. The hilar bile duct stricture was 3 cm in length. Intraductal ultrasonography (IDUS) did not detect any malignant cells in the bile duct around the hilar stricture, and no cancer cells were found in the endoscopic biopsy specimen. MDCT demonstrated a left portal vein obstruction, probably due to the inadvertent puncture of PTBD performed at the local hospital. Overall, no findings that indicated cholangiocarcinoma were observed. However, we determined that surgery was needed to treat this complicated biliary stricture. Left hepatectomy with caudate lobectomy and extrahepatic bile duct resection was performed. Severe adhesion around the hepatoduodenal ligament resulted in a difficult surgery. Intraoperative frozen section examination revealed no malignancy of the resected common bile duct stump and biliary stricture. The stumps of the resected B6 and the right hepatic duct were reconstructed by Roux-en-Y cholangiojejunostomy (Fig. 3c). The operative time was 591 min, and blood loss was 1745 ml.

**Fig. 4:**
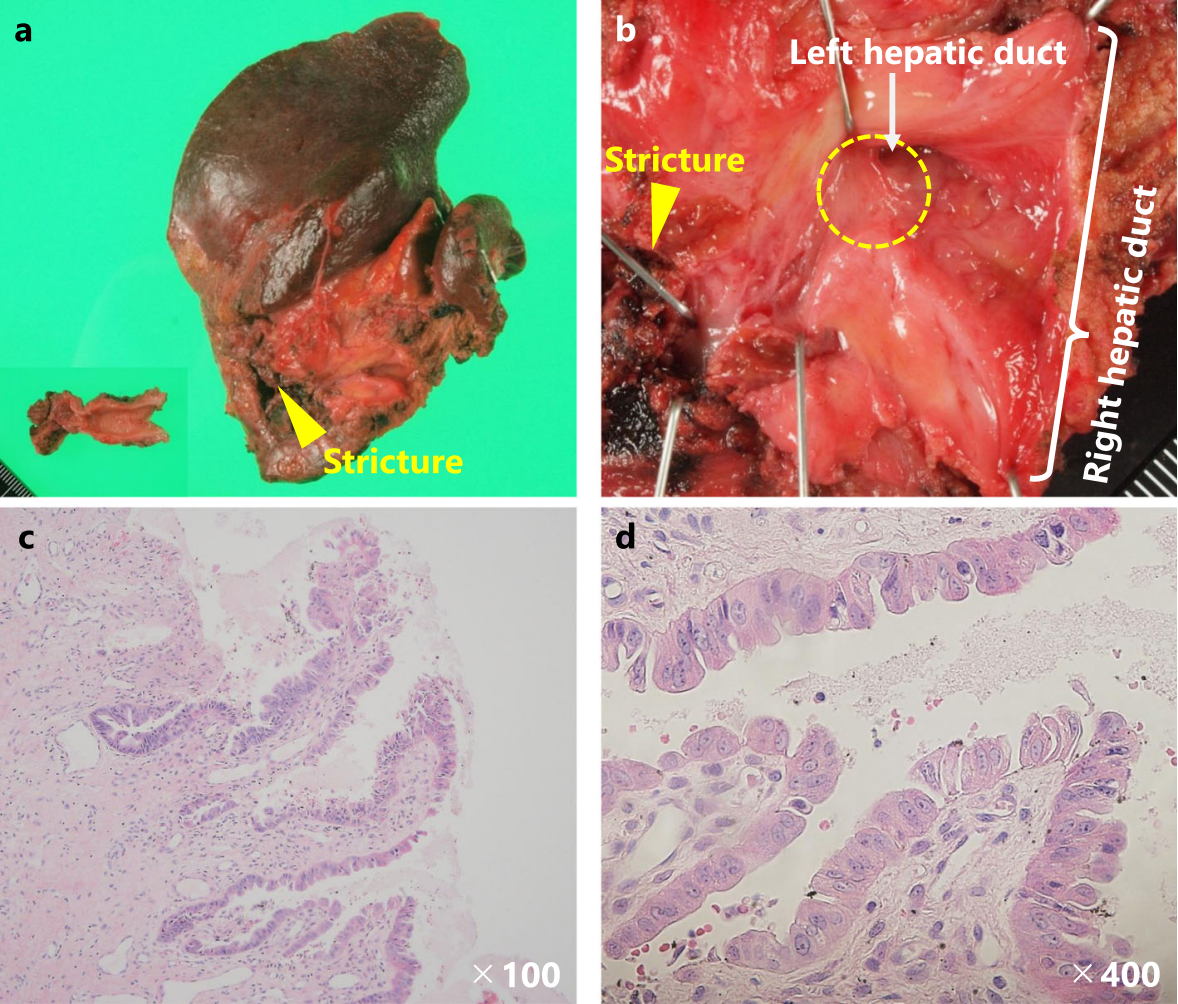
Macroscopic and microscopic findings of specimens. **a** Resected specimen. **b** Flat type in situ carcinoma located near the hepatic confluence (the yellow dotted circle). The carcinoma is distant from the bile duct stricture. **c**, **d** Microscopic findings of in situ carcinoma

The postoperative course was uneventful, and the patient was discharged on postoperative day 12. In the final pathology, no malignant cells were observed in the hilar stricture, but flat type in situ carcinoma was found at the confluence of the right and left hepatic ducts, which was distant from the stricture (Fig. 4). Histologically, the carcinoma was a well-differentiated adenocarcinoma and 1 cm in diameter. No lymph node metastases were found, and all of the surgical margins were negative. There were no BilIN lesions in the background bile duct. The patient is still alive and in good health 54 months after the hepatectomy.

## Discussion

When arterial blood flow to the bile duct is restricted, a bile duct stricture will develop [1, 2]. Several authors have reported delayed bile duct strictures due to ischemia after excision of the tissue surrounding the biliary tree [3–5]. Ishizuka et al. [3] reported two cases of delayed ischemic biliary stricture after radical lymphadenectomy in the hepatoduodenal ligament with skeletonization of the extrahepatic bile ducts for malignant diseases: in both cases, histologic examination of the subsequently resected biliary strictures revealed evidence of ischemia. Skeletonization of the extrahepatic bile duct may induce ischemia then delayed stricture formation. Lymphadenectomy of the hepatoduodenal ligament is routinely performed in advanced gallbladder carcinoma. However, when preserving the extrahepatic bile duct, this surgical procedure may induce bile duct stricture, and the present case is a case of delayed ischemic stricture. For prevention of this complication, assessment of arterial perfusion in the bile duct wall using indocyanine green near-infrared imaging may be a promising way [5].

Flat type precursor lesions are called biliary intraepithelial neoplasias and are classified into three grades [6]. Especially with severe atypia, the lesions are identical to in situ carcinoma. This kind of epithelial carcinoma is often detected as a lesion accompanied by invasive carcinoma [7], while in situ carcinoma alone is rarely detected. According to our previous study [8], only 3 cases of in situ carcinoma were found in 545 consecutive resections of perihilar cholangiocarcinoma. Thus, the incidence of in situ perihilar cholangiocarcinoma was 0.55%.

In this case, aspiration bile cytology alone showed a positive result. We performed aspiration cytology from the PTBD catheter five times, which was positive three times. Tsuchiya et al. [9] reported that the positivity of aspiration bile cytology increased when repeatedly performed but reached a plateau after five examinations. Aspiration cytology is easy to perform and repeatable [10]; thus, it should be used, especially in cases of negative biopsy results. However, aspiration cytology never indicates location of the lesion, which is a major limitation of this examination.

## Conclusions

We experienced a rare case of flat type in situ perihilar cholangiocarcinoma that was incidentally accompanied by a benign bile duct stricture. When the tumor is undetectable but aspiration cytology is positive, in situ cholangiocarcinoma may exist; thus, surgery should be carefully considered.

## Data Availability

The authors declare that all data in this article are available within this published article.

## Abbreviations

CBD: Common bile duct
ENBD: Endoscopic nasobiliary drainage
IDUS: Intraductal ultrasonography
MDCT: Multidetector-row computed tomography
PTBD: Percutaneous transhepatic biliary drainage
PTCS: Percutaneous transhepatic cholangioscopy
